# Psychogeriatrics in a world with COVID-19

**DOI:** 10.1017/S104161022000126X

**Published:** 2020-10

**Authors:** Mary Sano, Maria I. Lapid, Manabu Ikeda, Raimundo Mateos, Huali Wang, William E. Reichman

**Affiliations:** 1 Icahn School of Medicine at Mount Sinai, New York, NY, USA; 2 Mayo Clinic Department of Psychiatry and Psychology, Rochester, MN, USA; 3 Department of Psychiatry, Osaka University, Ibaraki-shi, Japan; 4 University of Santiago de Compostela, Santiago de Compostela, Spain; 5 Peking University Institute of Mental Health, Beijing, China; 6 Faculty of Medicine at the University of Toronto, Toronto, Canada

## Introduction

By the time this edition of our journal reaches our readers, we will have had many months to consider the meaning of a global pandemic to the patients we serve and to the diverse workforce of professionals comprising our field. The pandemic has compelled us to gain additional insights into what motivates each of us to serve our fellow citizens. For many of our colleagues around the world, the impact of coronavirus disease 2019 (COVID-19) has resulted in an even greater appreciation of the emotional, cognitive, and physical vulnerabilities of those we care for. In this introduction to a groundbreaking edition of the IPA journal, we have had the privilege to speak to colleagues from our own communities and across the globe. Our perspectives as practitioners in the field of psychogeriatrics have often been as a protected and privileged class, highly educated, well thought of, and likely to have strong ideas about how to lead and what to do next. Yet, we have been humbled by the recognition of exactly how little control we have to change a world that has been so profoundly affected by a disease which we know so little about.

The particular risk to older individuals has been recognized since we began tracking the devastation of COVID-19, with the highest reported mortality and most severe morbidity in those over age 65. Additionally, the cohort that we serve is highly likely to have comorbid conditions that increase the risk of especially adverse outcomes of this disease.

This COVID-19 experiential collection ncludes contributions from North America (United States, Canada), South America (Brazil), Caribbean (Cuba, Dominican Republic, and Puerto Rico), Europe (Belgium, Germany, Ireland, Italy, Netherlands, Norway, Portugal, Spain, and United Kingdom), Asia (China, Hongkong, Japan, Philippines, and Taiwan), Middle East (Lebanon), Israel, and Australia. Colleagues from around the world provide insights and unique perspectives on the impact of COVID-19 on the full spectrum of geriatric mental health. We hope this collective experience will inform our worldwide community of clinicians, educators, and researchers in ensuring the continuity of the International Psychogeriatric Association (IPA) mission to promote better mental health for older people around the world.

## Voices from the field

*United States*. Writing from New York City, the US epicenter for COVID-19, several colleagues, serving older patients in many different clinical settings, have openly shared their most challenging experiences, thoughts, and feelings. Their reflections are informative, sobering, and at times, remarkably inspiring.

In the hospital psychiatry practice, social distancing and visitation restrictions to prevent disease transmission have adversely affected hospitalized older individuals and led to worsening emotional distress, depression, anxiety, and a sense of enduring helplessness. Many patients were not able to comply with masking, handwashing, and social distancing. Persons with dementia, particularly those with COVID-19 disease, were prone to developing delirium, agitation, and behavioral problems. This was seemingly worsened by restrictive measures put in place to limit the spread of the virus. Social distancing and infection control measures have prevented the use of shared spaces and group activities. This has led to increased social isolation and fewer psychotherapeutic interventions being available to patients.

The psychiatric Emergency Department (ED) practice had also been transformed into “a battle field” in which the invading enemy was an infectious disease. Previous consultation rooms were converted into COVID-19 units, where assessment and management are conducted in small physical spaces. Some patients refuse to wear masks, making the provision of emergency care especially difficult. Ethically challenging decisions must be continuously made as to who gets admitted because of bed shortages and the pressure to get patients discharged from the ED. Homeless patients with erratic behaviors and baseline poor judgment, presenting a special risk for spreading contagion to others, may not warrant hospitalization and, therefore, get discharged from the ED back to the streets. The impossible choices became everyday events. Psychiatrists were also expected to conduct telepsychiatry consultations to the ED and medical floors, or if not available, to review the chart and provide the best advice, remotely. A geriatric psychiatrist wrote “I will never know if the patient with vascular dementia and was post-op from surgical amputation for gangrene was trembling from alcohol-withdrawal or myoclonic jerks in delirium.”

Electroconvulsive therapy (ECT) availability and practice also drastically changed. As the number of COVID-19 cases grew and the death count rose, hospitals feared over capacity, and all elective procedures had been suspended. Although ECT is deemed an essential treatment (Practice Guidance for COVID-19, 2020), especially for older individuals with severe or treatment-resistant psychiatric symptoms, the lack of anesthesia and nursing staff due to redeployment led to a reduction of ECT services and an increased risk of clinical deterioration of our older patients.

Across the clinical settings, staff received daily critical updates on the latest in personnel protective equipment (PPE) advice, the location of telehealth machines in the intensive care unit (ICU), and emergent coverage needs. The ability to head in and try to help colleagues, patients, and families in any way provided some diversion from the constant fear. The stories of the chaos in the hospital wards, ICUs, and EDs were plastered throughout the city in every form of media and created panic and fear for many older people. One devastating impact of the disease was its forced separation of families so that healthcare providers often needed to make urgent life or death decisions, without the ability to get input from older patients and their loved ones.

These stories of patients in healthcare systems without access to friends and families, in which medical conditions rapidly deteriorate with a high likelihood of adverse outcomes, remind us of the importance of end of life care. COVID-19 in the elderly is not uniformly fatal; however, it is frequently associated with severe outcomes such as hospitalizations and ICU admissions resulting in high mortality (CDC COVID-19 Response Team, 2020). The possibility of facing a life-threatening illness such as COVID-19 alone when one is unable to decide or speak for themselves, makes it imperative for our patients to have advance care planning in place in order to ensure their wishes and preferences for medical care are known to their loved ones and to medical providers.

*Canada*. The Canadian hospital experience is a bit different from that of the USA (Flint *et al.*, 2020). The universal healthcare system has minimized the financial impact on older Canadians’ access to health care. The country’s response to the pandemic is informed by previous experience with the severe acute respiratory syndrome coronavirus 1 outbreak that led to a better public health system and more preparedness for disease outbreaks and epidemics. This has limited the burden on hospital systems and mitigated any potential major disruption to the delivery of mental health care. However, elective medical admissions and surgical procedures were suspended during the pandemic as most ambulatory services were deemed non-critical.

Most importantly, the pandemic has exposed very serious problems and vulnerabilities in Canada’s Long-Term Care (LTC) facilities. Approximately, 80% of the country’s COVID-19-related deaths have occurred in these settings. Many were wholly unprepared for rapidly evolving outbreaks of infectious disease. There was a lack of adequate supplies of PPE, undertrained staff, too many residents sharing bedrooms and common spaces in close proximity to each other. Social distancing was nearly impossible in many older facilities and residents with dementia, who were prone to wander, presented a special risk. Social distancing measures prohibited family visitation to LTC facilities across the country. Many staff members had been working part time in a variety of different nursing homes and were thus inadvertent vectors for COVID-19 spread. In some homes with especially severe outbreaks, staff refused to come to work. In the extreme cases, the Army was summoned by the government to go in to these facilities to provide care. In others, all of the residents were transferred to local hospitals.

*Italy*. In Italy, the lack of preparedness for a pandemic devastated the country and resulted in the loss of many lives (De Leo and Trabucchi, 2020). The older adult population was disproportionately affected by COVID-19 infections and deaths. Geriatric mental health was neglected, and the persistent loneliness, forced isolation, and fear of contracting the illness increased the risks of unmet psychological needs in older adults.

*China*. In China, people aged 60 years and older comprised more than one-third of COVID-19 cases and more than 80% of deaths (Wang *et al.*, 2020). Older people were more vulnerable to anxiety, stress, sleep difficulties, agitation, and behavioral problems. The commentary in this special issue describes a coordinated national response through a collaboration of professional psychiatric, geriatric, and other interdisciplinary societies to address the mental health needs of older people and their caregivers in a timely manner.

*Japan*. In Japan, even before general healthcare services were overwhelmed, the care systems specifically designed for older adults were already in collapse. As of April 23, 2020, only 10,581 cases and 293 deaths were reported yet, at least 883-day service programs for elders were closed across the country. Family caregivers had stopped their elderly relatives from using these services to reduce the risk of infection posed by attending senior care facilities. In communal environments serving older people with dementia and supporting family caregivers, their quality of life has seemingly worsened on a daily basis. Older patients who have been cut off from accessing outpatient rehabilitation have been noted to experience an acute deterioration in behavioral and psychological symptoms of dementia. In several cases, this tragic consequence has resulted in abusive behavior directed toward elders by caregiving family members. Under these very trying circumstances, Japan’s mental health specialists for older adults have been expected to provide full support to patients and their families, and the staff members of care facilities. It has been a great challenge.

*Philippines*. In the Philippines, a developing country with limited resources, and an inadequate healthcare infrastructure, vulnerability to the negative impacts of COVID-19 are underscored (Buenaventura *et al.*, 2020). During the pandemic, chronic challenges in meeting the most basic health needs are magnified, and there are no mechanisms in place to meet the mental health needs of the older population.

## Technology to the rescue, maybe?

The COVID-19 pandemic has accelerated the use of technology across the globe. In the new norm of social distancing, technology has provided older individuals a connection with the real world, a means to communicate with friends and family, and a way to overcome loneliness and isolation. However, availability and access to technology are limited to those jurisdictions and those citizens with adequate financial resources. Older age, lower income, less education, and geographic remoteness all lead to more reduced access to technology. Many older individuals are not technology savvy or lack functional mobile phones. For individuals with dementia or milder forms of cognitive impairment, using a computer or smartphone may be especially challenging. In addition, few telelinks have high-quality captioning, which is a must for those who have disabling hearing loss that affects nearly 25% of those aged 65 to 74 and 50% of those who are 75 and older (NIDCD, 2020). Telehealth has transformed clinical practice in this pandemic and will have a long-lasting impact on geriatric mental healthcare delivery beyond COVID-19. It holds great promise in providing care to patients and their caregivers that is convenient, accessible, and affordable. However, for telepsychiatry to be useful for older individuals, further technological improvements are needed to accommodate age-related sensory and cognitive impairments.

## Impact on psychogeriatric education

The field of geriatric mental health has always suffered from an insufficient workforce with few certification programs for geriatric psychiatry worldwide (Wang *et al.*, 2013). The same inadequate level of geriatric specialization programs plagues all collaborating disciplines, including psychology, nursing, social work, and physical therapy.

In this time of COVID-19, all training has suffered. Faculty members have to prioritize clinical duties that take time away from education. The interruption of the training of learners at all levels has negatively impacted their education and career progression. Students in distance learning environments miss out on direct observations and interactions in the classroom and bedside. Educators confined to teaching virtually have no opportunity to observe learners’ social dynamics or identify who might need more encouragement or attention. Travel restrictions and cancelation of medical conferences have hampered the personal and professional development of learners. Some medical schools and graduate medical education programs in the USA have accelerated graduations in order to increase the workforce where there are shortages. In other areas of the world, student practitioners have left school to be deployed to high medical needs, missing the opportunity to ensure critical competencies.

Education is extensively affected by the COVID-19 pandemic at the individual, institutional, and global levels. With learners making up a significant portion of the workforce, educational efforts across the globe will need to focus on the preservation of education to protect learners while still ensuring their emotional, physical, and psychosocial well-being. It is unclear to what extent this negative impact on education will affect geriatric mental health care, and this needs to be closely monitored. With the anticipation that the COVID-19 outbreak will last a long time and its consequence of growing mental health problems, especially in older individuals, it is more critical than ever to ensure a supply of trained, skilled, and expert providers in providing geriatric mental health care.

## Impact on research in geriatric mental health

The social distancing measures put in place during the pandemic have had a substantially adverse impact on most research programs in our field. Work from home mandates and the limitation on non-essential visitation to healthcare facilities have greatly hampered clinical and even, non-clinical research to continue. Human subject volunteers cannot be recruited, and they cannot come to hospital or university centers to participate in research given travel and other pandemic-related restrictions. In some places, hospital-based research assistants and other research staff have been redeployed to support COVID-related activities in the healthcare settings. Many research activities have been suspended as a result, and some may not be able to be restarted at all.

## Impact on ageism

The recognition that people aged 65 years and older who have underlying multiple medical comorbidities and live in long-term care settings are at higher risk for severe illness and mortality from COVID-19 has inadvertently caused the reemergence of ageism.^1^ Using age to separate at-risk individuals promotes negative stereotypes and ignores the majority of older people who are healthy and well. The use of age and life years saved to determine scarce resource allocation is discriminatory and is stigmatizing against older adults. The commentary on ageism in this special issue emphasizes that chronological age should not be a sole criterion for determining risk, prognosis, or treatment options, nor should it be used for resource allocation (Ayalon, 2020). The commentary describes strategies to combat ageism in the COVID-19 era, consistent with United Nations policy on protecting the human rights of older persons.^2^

## IPA stands to serve in times of a pandemic

IPA fundamentally exists to support the work of all of us who are devoted to the care of older adults and to specifically promote geriatric mental health across the globe through education, research, professional development, advocacy, health promotion, and the introduction of innovative models of care. Vitally, the pandemic has intensified the need for our field to effectively promote broader respect for the needs and rights of older persons and to advocate for better access to care in settings that are accessible, compassionate, safe and respectful of personal desires and wishes. At the beginning of this century, IPA was one of the key international entities, under the WHO’s aegis, that led to the conceptualization of principles of psychogeriatric care (World Health Organization, 1997). While the pandemic presents significant challenges to all of us, it should also provide opportunities to advance the effectiveness of our life’s work. We are hopeful that through this special Journal edition and our related IPA pandemic focused activities, our shared experience of the effects of COVID-19 will lead to better care for all of those to whose well-being we are collectively devoted.

## Footnotes

1 https://www.cdc.gov/coronavirus/2019-ncov/need-extra-precautions/peopleat-higher-risk.html

2 https://unsdg.un.org/sites/default/files/2020-05/Policy-Brief-The-Impact-of-COVID-19-on-Older-Persons.pdf, accessed May 30, 2020.
